# Audio recordings dataset of grazing jaw movements in dairy cattle

**DOI:** 10.1016/j.dib.2020.105623

**Published:** 2020-04-30

**Authors:** Sebastián R. Vanrell, José O. Chelotti, Leandro A. Bugnon, H. Leonardo Rufiner, Diego H. Milone, Emilio A. Laca, Julio R. Galli

**Affiliations:** a Research Institute for Signals, Systems and Computational Intelligence, sinc(i) (FICH-UNL/CONICET), Ciudad Universitaria, Santa Fe, Argentina; b Department of Plant Sciences, University of California, Davis, USA; c Facultad de Ciencias Agrarias, Universidad Nacional de Rosario, Zavalla, Argentina; d Instituto de Investigaciones en Ciencias Agrarias de Rosario, IICAR (UNR/CONICET), Zavalla, Argentina; e Laboratorio de Cibernética, Facultad de Ingeniería, Universidad Nacional de Entre Ríos, Oro Verde, Argentina

**Keywords:** Cattle grazing behavior, Ingestive behavior, Acoustic monitoring, Signal processing, Feature extraction

## Abstract

This dataset is composed of correlated audio recordings and labels of ingestive jaw movements performed during grazing by dairy cattle. Using a wireless microphone, we recorded sounds of three Holstein dairy cows grazing short and tall alfalfa and short and tall fescue. Two experts in grazing behavior identified and labeled the start, end, and type of each jaw movement: bite, chew, and chew-bite (compound movement). For each segment of raw audio corresponding to a jaw movement we computed four well-known features: amplitude, duration, zero crossings, and envelope symmetry. These features are in the dataset and can be used as inputs to build automated methods for classification of ingestive jaw movements. Cow's grazing behavior can be monitored and characterized by identifying and analyzing these masticatory events.

Specifications Table

**Table:** 

**Subject**	Animal Science / Computer Science
**Specific subject area**	Bioacoustic foraging monitoring / Signal processing
**Type of data**	Audio recordings (WAV, mono, 16-bits, 22.05 kHz) Jaw-movement labels (TXT, tabular separated values) Jaw-movement features (CSV)
**How data were acquired**	Audio recordings were acquired using a wireless microphone (Nady 151 VR, Nady Systems, Oakland, CA, USA) Two experts in animal behavior identified events and added labels for start, end and type Features file was obtained by processing raw audio recordings
**Data format**	Raw, processed, analyzed
**Parameters for data collection**	Cows grazing two forage species (pure alfalfa or pure tall fescue), each at two heights (tall and short)
**Description of data collection**	Audios were recorded on three cows grazing on two forage species in individual grazing sessions: pure alfalfa or pure fescue microswards at two heights (tall or short)
**Data source location**	Institution: Campo Experimental J.F. Villarino, Facultad de Ciencias Agrarias, Universidad Nacional de Rosario City: Zavalla Province: Santa Fe Country: Argentina
**Data accessibility**	Direct URL to data: https://github.com/sinc-lab/dataset-jaw-movements

## Value of the data

• We provide a fully labeled dataset of cattle grazing behavior comprising raw audios, labels for jaw movement, and extracted features.
• To the best of our knowledge, this is the first dataset on acoustic monitoring of ruminant behavior fully available to the scientific community.
• Jaw-movement recognition is the basis for studying nutrition, forage intake and welfare of ruminant livestock.
• This dataset provides audio recordings of the ingestion of two pastures (alfalfa and fescue), each at two heights (short and tall).
• The dataset can be used to develop signal processing and machine learning methods for jaw-movement detection, segmentation, and classification.

## 1 Data Description

Data consist of a set of 52 audio files of the recorded ingestive sounds made by dairy cattle grazing all combinations of short and tall alfalfa and fescue, 52 TXT label files corresponding to those audio files, and a single CSV summary file. TXT label files contain the results of segmentation and classification of sounds into bites, chews and chew-bites conducted by two experts for all audio files, with the aid of video records. The CSV file condenses in a single tabular format the data and metadata for each jaw movement in the dataset, comprising recording metadata, segmentation, classification, and extracted features.

Accurate monitoring of animal foraging behavior is a complex but essential task to optimize livestock production systems [1]. Changes in ruminant foraging behavior are indicators of animal health and welfare and can be useful in early detection and prevention of several diseases. Precision livestock farming is a useful approach to tackle these problems using advanced technology to monitor each animal individually. In particular, acoustic monitoring is reliable to recognize and quantify jaw movements (JM) in free-ranging cattle [2], [3], [4], [5], [6].

In a short timescale, foraging behavior of ruminants can be characterized by JM, which have a duration close to 1 s. The JM are: biting, when herbage is apprehended and severed; chewing, when herbage is comminuted; and a combination of chewing and biting in a single movement, called chew-bite [7,8]. The grazing process involves searching, apprehending, chewing, and swallowing herbage. During grazing, JM are performed regularly with a frequency that ranges from 0.75 to 1.20 JM/s [9].

A summary of the dataset contents is in Table 1. There are 52 audio files, recorded on a set of three Holstein dairy cows grazing two pastures (alfalfa or tall fescue), each at two heights (short or tall). Each audio file (e.g. recording_23.wav) has a corresponding label file (e.g. recording_23.txt), containing the segmentation (start and end), and the classification label of all the JM in the recording. Each JM can be one of three categories: bite, chew, or chew-bite. Audio and corresponding label files are contained in the *audios* and *labels* folders, respectively.

**Table 1:** Summary of audio files grouped by pasture and height.

Pasture	Height	Files (filename id number)	Chews	Bites	Chew-Bites	Overall Duration
Alfalfa	Tall	17 (01 to 17)	416	148	322	14 min 26 s
Alfalfa	Short	11 (18 to 28)	260	179	123	12 min 42 s
Tall fescue	Tall	12 (41 to 52)	487	100	238	14 min 03 s
Tall fescue	Short	12 (29 to 40)	454	94	217	13 min 13 s
**Total**		52	1617	521	900	54 min 24 s

**Fig. 1:**
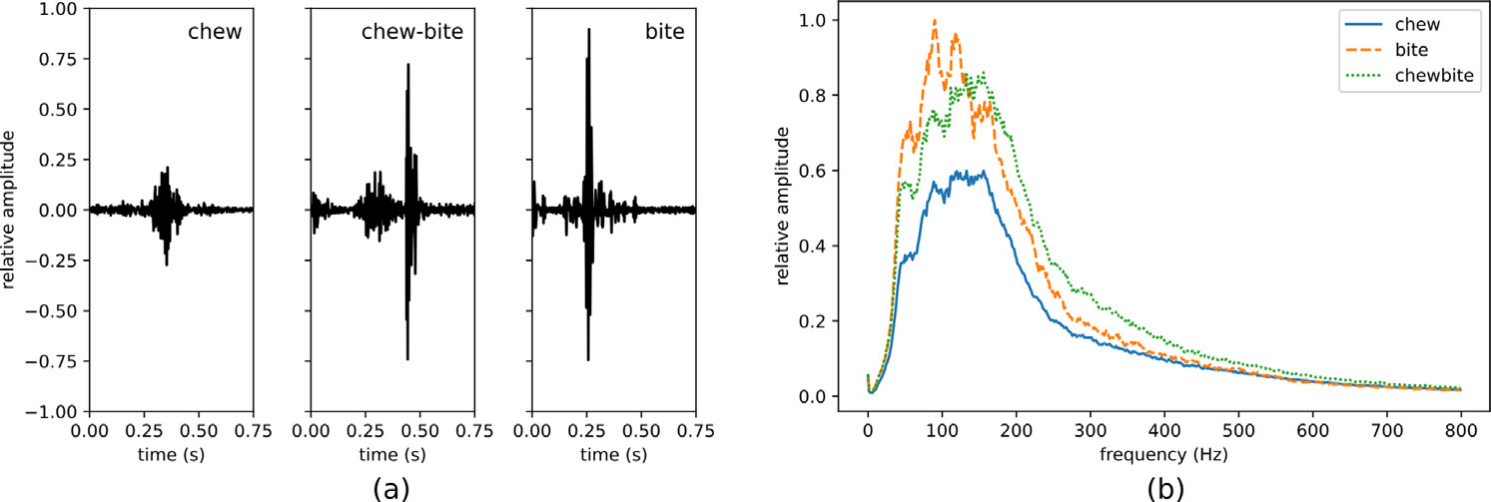
The three types of jaw movements. (a) Typical sound waveform. (b) Average spectra on tall alfalfa.

Typical waveforms and spectra for the three types of JM are in Fig. 1. Bites and chews have different wave morphologies, while chew-bites are a combination of both. The average spectra of the three jaw movements in tall alfalfa have similarities, but the energy of chews is smaller than that of bites and chew-bites.

We computed four well-known features of the audio signal of each labeled JM [2]. The features, along with recording metadata, are contained in a single CSV file: features.csv. Each row of the file corresponds to one JM. The columns in this file are:

• **zero crossings:** number of zero crossings for the derivative of the envelope signal
• **amplitude:** the maximum absolute value of the segmented movement in audio signal
• **duration:** length of the JM, computed on the envelope signal (given in seconds)
• **envelope symmetry:** a symmetry measure on the envelope signal corresponding to a JM
• **label:** type of JM (chew, bite, or chew-bite)
• **start:** time where each JM starts (given in seconds)
• **end:** time where each JM ends (given in seconds)
• **species:** pasture species (alfalfa or tall fescue)
• **height:** pasture height (short or tall)
• **recording:** name of the audio file containing the JM

## 2 Experimental Design, Materials and Methods

**Fig. 2:**
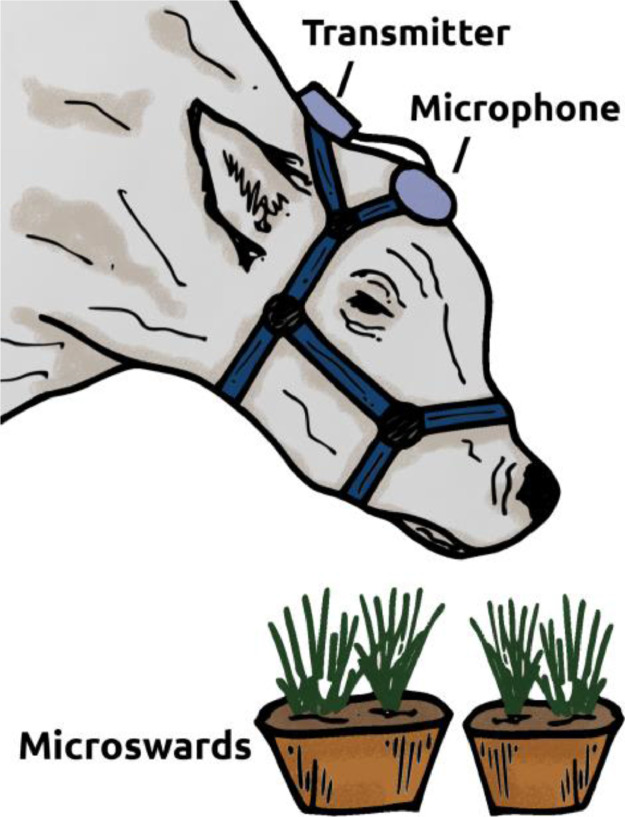
Microphone was located facing inwards on the forehead of the cow and the transmitter was placed on the neck. The cow is grazing microswards.

The fieldwork to obtain this dataset took place at the Campo Experimental J.F. Villarino, Facultad de Ciencias Agrarias, Universidad Nacional de Rosario, Zavalla, Argentina. The Committee on Ethical Use of Animals for Research of the Universidad Nacional de Rosario evaluated and approved project protocols. We recorded ingestive sounds produced by dairy cows in individual grazing sessions conducted over a 5-day period. Microswards consisted of sets of 4-liter plastic pots with either alfalfa (*Medicago sativa*) or tall fescue (*Lolium arundinaceum*, Schreb.), tall (24.5 ± 3.8 cm) or short (11.6 ± 1.9 cm), firmly attached to the floor. Each of three 4–6 year-old lactating Holstein cows weighing 608 ± 24.9 kg grazed all four microsward types, one cow and microsward per recording session. Cows were tame and trained in the experimental routine. Each day we randomly assigned one of the three microphones (Nady 151 VR, Nady Systems, Oakland, CA, USA) to each cow, placed it facing inwards on the cow's forehead and covered it with rubber foam [10] (Fig. 2). The distance between the wireless transmitter and receiver was 2-3 m. We recorded video and sound of cows grazing with an analog video camcorder to assist the labeling by the experts. A standard beeping sound (frequency: 4100 Hz) was produced and recorded every 10 s to equalize sound intensity across recordings. Even though the recordings were obtained indoors, some of them contain different types of environmental noises, such as birdsongs. More details can be found in [5].

We provide raw audio signals from the video soundtrack as WAV audio files (mono, 16-bits, 22.05 kHz). Audio signals consist of sequences of events – bites, chews, and chew-bites – separated by silences and environmental noises. Experts in ruminant grazing behavior, well trained in recognition of ingestive sounds, viewed the video tapes and listened to the recordings to accurately identify each JM (start, end, and label) on the plot of the sound waveform. First, one of the experts labeled the signals, and then, the results were checked by the other expert. Detections agreed 100% for bites, 98.2% for chews, and 99.1% for chewbites. There were 2.7% of insertions and 0.9% of deletions. Thus, the total segmentation and classification accuracy was 93.6%. Experts worked together to achieve a final decision in case of disagreement.

Prior to feature extraction, we pre-processed each raw audio signal by applying a least mean square filter to remove trends or low-frequency noises [11]. Then, the pre-processed signal was decimated to 2 kHz and the *amplitude* was computed for each labeled JM. We obtained the envelope of the signal by applying a low-pass filter to the previously decimated signal. The rest of the features (i.e. *duration, zero-crossing*, and *envelope symmetry*) were extracted from the envelope of the signal for each labeled JM. More details on the features computation are in [2].

**Fig. 3:**
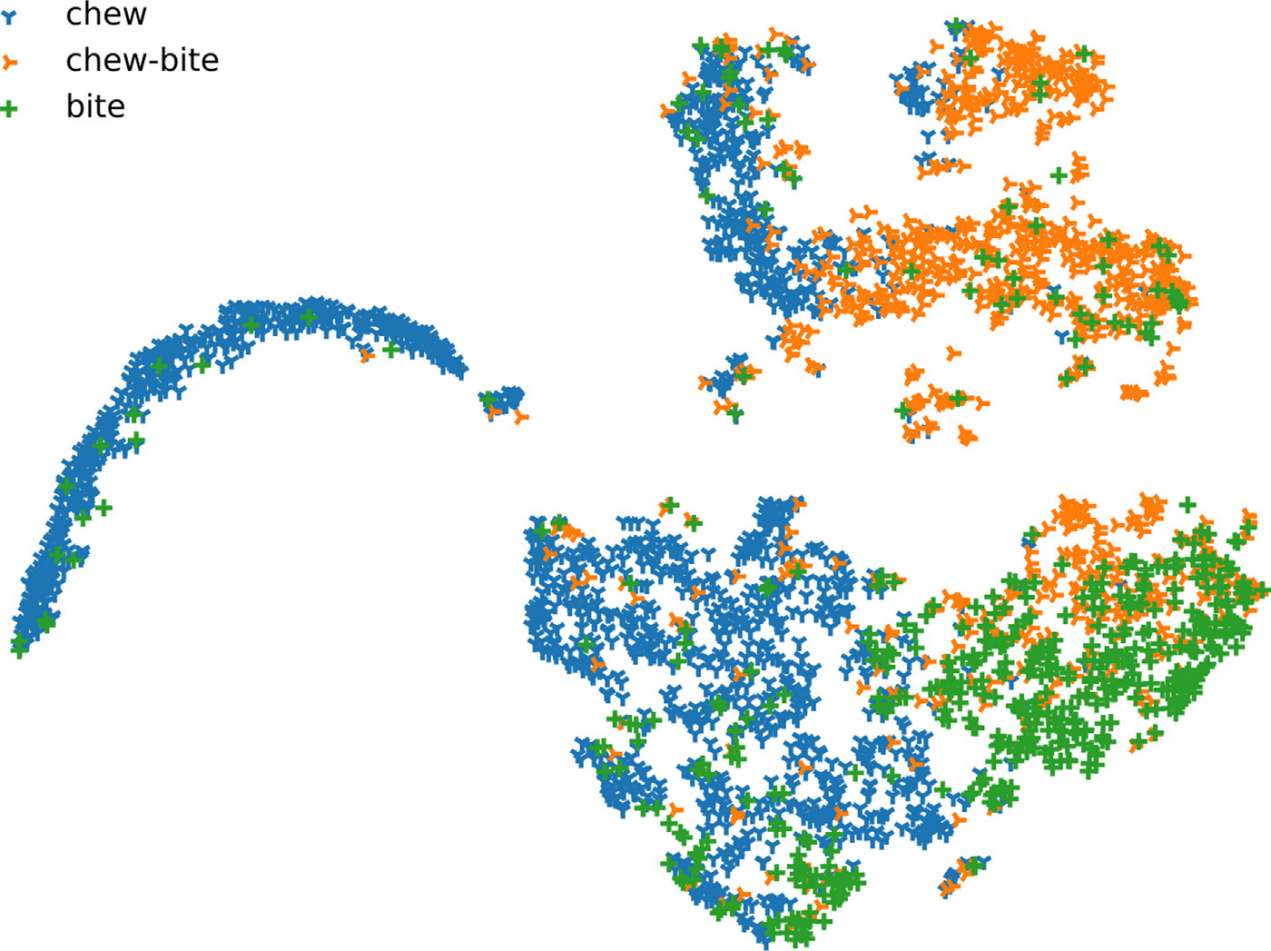
T-distributed stochastic neighbor embedding of the complete feature set. Each data point corresponds to a jaw movement.

**Fig. 4:**
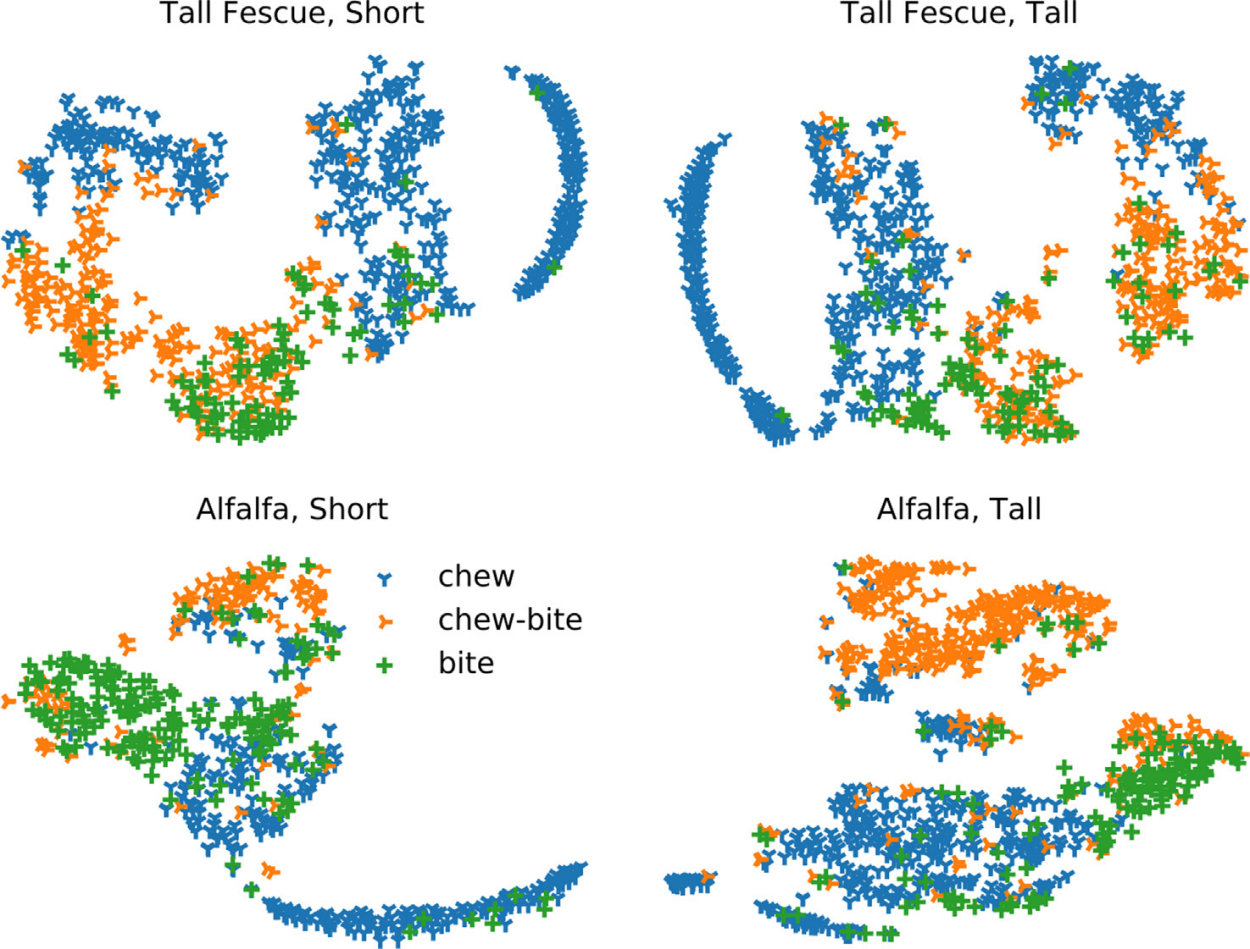
T-distributed stochastic neighbor embedding for datasets per species and height. Each data point corresponds to a jaw movement.

We used a dimensionality reduction method to visualize the features of events. Each data point is a single JM in the feature space. T-distributed stochastic neighbor embedding (t-SNE) was generated to further describe the provided features [12]. In Fig. 3, all samples are embedded into the same space. Points are grouped closely for each class, showing the discriminative power of these features. Fig. 4 shows t-SNE plots for each combination of species and height, giving four different embedded spaces. Interclass relationships are similar to Fig. 3.

**Fig. 5:**
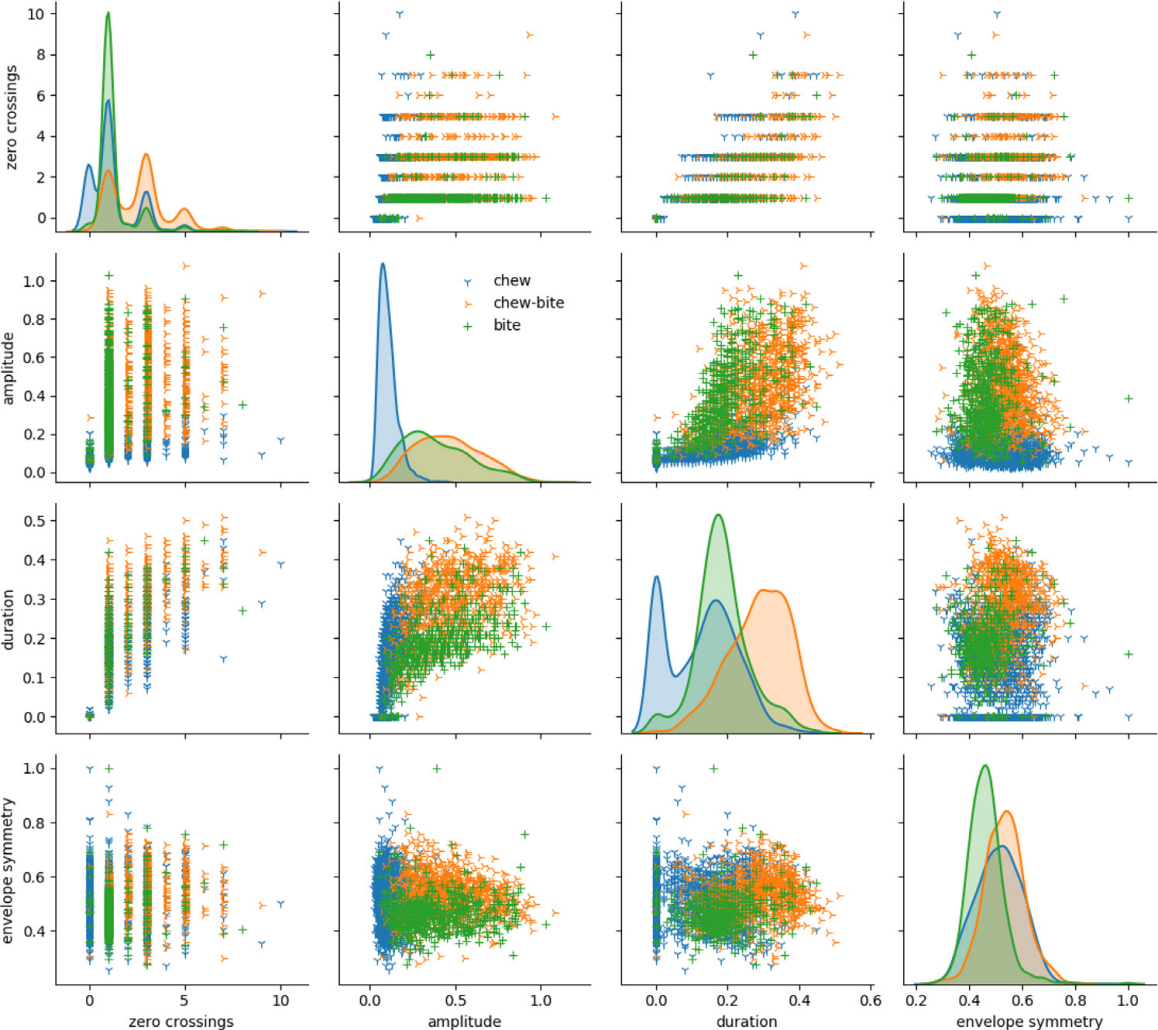
Uni- and bivariate distributions of features for all species and heights by jaw movement type.

Fig. 5 shows a pair-plot of the four features. The plots in the main diagonal of the matrix are the kernel-smoothed densities of each feature, for each type of JM. Off diagonal panels show bivariate distributions. Amplitude-duration and amplitude-envelope symmetry plots exhibit good clustering by JM type.

## Appendix Supplementary materials

Image, application 1
